# Acid-sensing ion channels: potential therapeutic targets for neurologic diseases

**DOI:** 10.1186/s40035-015-0031-3

**Published:** 2015-05-30

**Authors:** Sha Liu, Xiao-Yu Cheng, Fen Wang, Chun-Feng Liu

**Affiliations:** Department of Neurology, the Second Affiliated Hospital of Soochow University, Soochow University, 1055 Sanxiang Road, Suzhou, 215004 China; Institute of Neuroscience, Soochow University, Suzhou, 215123 China; Beijing Key Laboratory for Parkinson’s Disease, Beijing, 100053 China

**Keywords:** Acid sensing ionic channels, Neurologic diseases, Pathophysiology, Neurodegeneration

## Abstract

Maintaining the physiological pH of interstitial fluid is crucial for normal cellular functions. In disease states, tissue acidosis is a common pathologic change causing abnormal activation of acid-sensing ion channels (ASICs), which according to cumulative evidence, significantly contributes to inflammation, mitochondrial dysfunction, and other pathologic mechanisms (i.e., pain, stroke, and psychiatric conditions). Thus, it has become increasingly clear that ASICs are critical in the progression of neurologic diseases. This review is focused on the importance of ASICs as potential therapeutic targets in combating neurologic diseases.

Under normal physiologic conditions, intra- and extracellular pH is maintained between 7.3-7.0. Increased neuronal excitability alters pH to activate various receptors and ion channels in cell membranes, including a variety of voltage-gated and ligand-gated ion channels [1, 2]. It has recently been shown that acid-sensing ion channels (ASICs) first cloned by Waldmann and colleagues [3] are activated in response to diminished extracellular pH [4]. However, the functional roles of ASICs in peripheral and central components of the nervous system remain unclear.

Neurodegenerative diseases, such as Parkinson’s disease, Huntington’s disease, and Alzheimer’s disease, are not curable at present. Although animal models of such conditions have been utilized to develop new therapeutic strategies, effective treatments have been elusive. Clinical trial and animal experimentation outcomes suggest that Ca^2+^ overload, mitochondrial dysfunction, oxidative stress, energy metabolism, and acidosis are involved in neurodegenerative processes. Consequently, ASICs may be pivotal in the pathophysiology of these disorders.

## Basic characteristics of ASICs

ASICs are ligand-gated cation channels activated by extracellular H^+^ and are widely distributed in mammalian central and peripheral nervous systems. As members of the degenerin and epithelial Na^+^ channel (DEG/ENaC) ion channel superfamily, displaying high Na^+^ permeability and sensitivity to amiloride blockade (with brief exposure), ASICs are composed of two hydrophobic transmembrane domains, short N- and C- termini, and a large extracellular loop (ECD) spanning the two hydrophobic domains [5–7]. The ECD harbors a so-called “acidic pocket” region that is responsible for acid-dependent gating, desensitization, and response to extracellular modulators [8]. This acidic pocket contains several pairs of acidic amino acids, is situated at the interface between two subunits, and is considered one of the ASIC pH sensors. Cations may gain access via lateral fenestrations in the wrist region and then move into a broad extracellular vestibule [8, 9]. The crystal structure of chicken ASIC1 incorporates a trimeric channel complex, marked by three radial subunits and a central ion pore [10]. Discovery of the ASIC crystal structure provided insight into inherent mechanisms of channel depolarization, inactivation, and modulation. The three-dimensional ECD consists of 12 b-sheets and 7 a-helices [9]. Two long a-helices form the transmembrane domains, which line the ion pore [9].

ASICs were first identified in the nervous system and cloned 14 years ago [11]. In mammals, there are six distinct ASIC subunits (ASIC1a, ASIC1b, ASIC2a, ASIC2b, ASIC3, and ASIC4) encoded by four different genes (*ACCN*1–4) (Table 1) [12]. These subunits form functional ion channels as dimers or heteropolymers, and differing channels vary in activation conditions, ion selectivity, kinetics, and distributions.

**Table 1:** Properties and distributions of ASIC subunits

ASIC subtype	pH sensitivity	Distribution
ASIC1a	5.8–6.8	CNS and PNS
ASIC1b	6.1–6.2	PNS
ASIC2a	4.5–4.9	CNS and PNS
ASIC2b	not applicable	CNS and PNS
ASIC3	6.4–6.6	PNS
ASIC4	not applicable	CNS and PNS

**Fig. 1:**
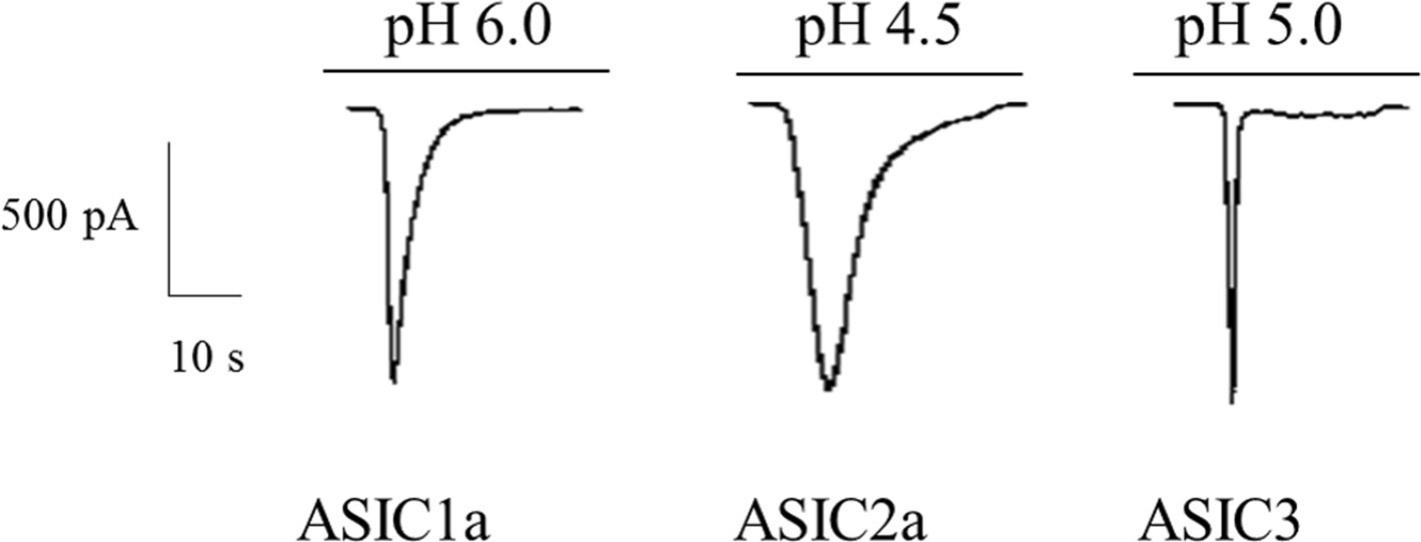
Biophysical properties of ASICs: representative current traces for ASIC1a, ASIC2a, and ASIC3 at pH 6.0, 4.5, and 5.0, respectively (membrane potential fixed at -60 mV)

**Fig. 2:**
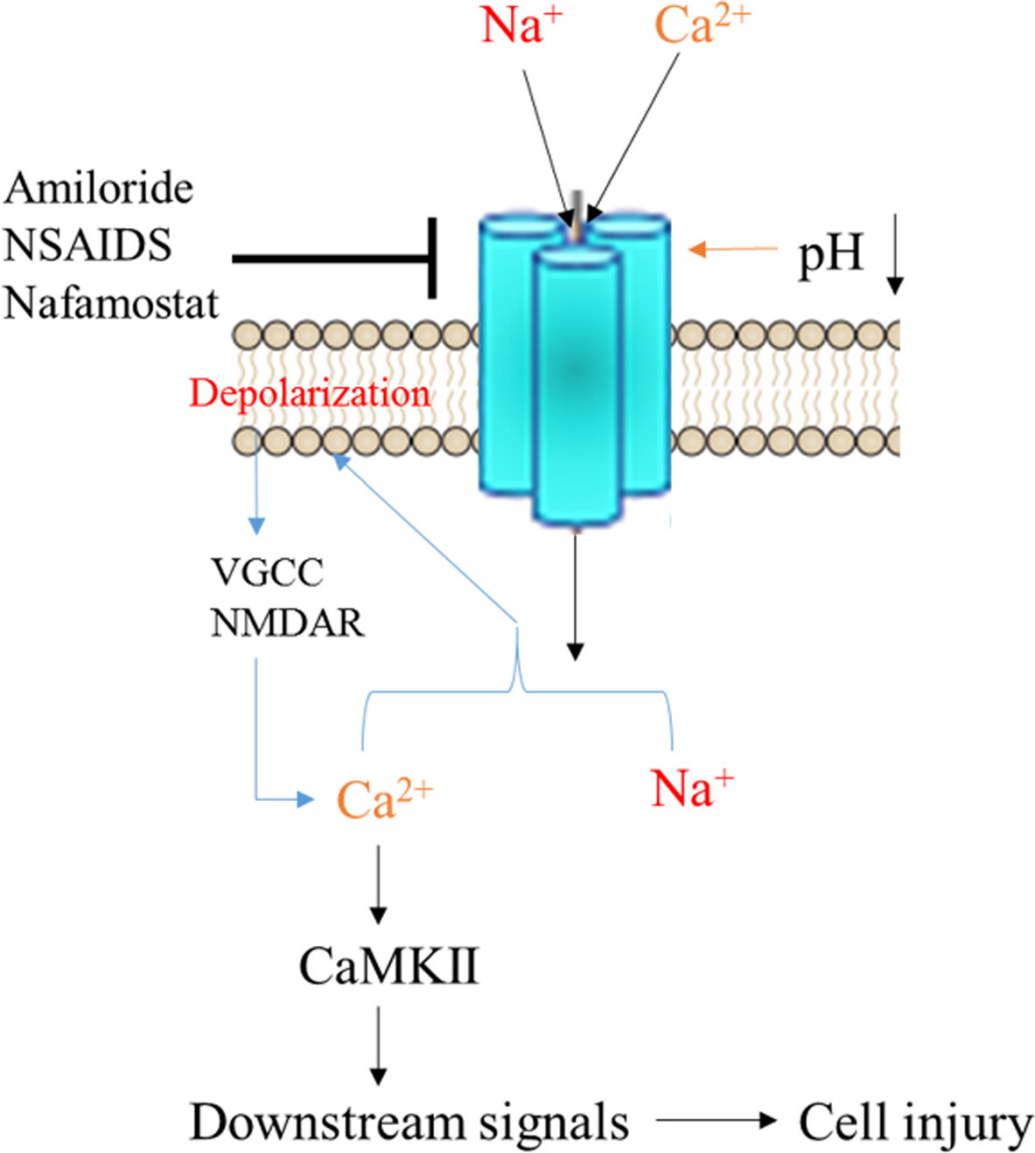
ASICs are activated by extracellular protons (H^+^) and are primarily permeable to Na^+^ (Ca^2+^ to a lesser degree). ASIC activation depolarizes cell membranes, activating voltage-gated Ca^2+^ channels (VGCCs) and N-methyl-D-aspartate receptors (NMDARs) to induce influx of Ca^2+^. Ca^2+^ influx contributes to calmodulin-dependent protein kinase II (CaMKII) phosphorylation and may influence other second-messenger pathways. Amiloride, NSAIDs, and nafamostat are ASIC inhibitors

ASICs are activated by falls in extracellular pH, particularly tissue acidosis (Fig. 1). Whole-cell patch clamp recording studies show that an increase in intracellular Ca^2+^ due to cerebral ischemia may be attenuated by the nonspecific Na^+^ channel inhibitor amiloride or the specific ASIC1a inhibitor PcTx1, both of which prevent tissue damage (Fig. 2) [13]. Other studies indicate that ASIC1a-mediated Ca^2+^ overload contributes to ischemic nerve cell death and inflammation in multiple sclerosis, with PcTx1 protecting cells by reducing Ca^2+^ influx [14]. Thus, local tissue acidosis induced by cerebral ischemia results in part from intracellular Ca^2+^ influx triggered by ASIC activation [15]. Still another study maintains that limiting ASIC1a expression after infarction exerts a neuroprotective effect [12]. Evidence shows that ASICs are involved sensations of pain and taste [16].

## Localization and electrophysiologic characteristics of ASICs

In order to understand the physiologic functions of ASICs, determining their distributions and electrophysiologic characteristics is critical. ASIC1a, ASIC1b, ASIC2a, and ASIC3 activate and induce inward current at pH values of 6.2, 5.9, 4.4, and 6.5, respectively [17]. In retinal ganglia, a decline in synaptic cleft pH inhibits the opening of presynaptic membrane Ca^2+^ channels. Changes in synaptic cleft pH likely are important in activation of ASICs [18]. ASIC1a is expressed at synapses, primarily in conjunction with the postsynaptic membrane [19]. Knock out of ASIC1a inhibits pH 5.0-induced inward current in cultured neurons from hippocampus, amygdala, and cortex [20]. ASIC1a is also co-expressed with ASIC1b in sensory neurons [21]. The ASIC2b subunit, which by itself is nonfunctional, is chiefly expressed in neurons of outer peripheral and central nervous systems, whereas ASIC3 is largely expressed in dorsal root ganglia (DRG) [22]. Other researchers have demonstrated expression of ASIC3 in bone, muscle, and cardiac tissue and expression of ASIC4 in pituitary gland [21, 23].

## Pharmacologic characteristics of ASICs

Amiloride, which is widely used clinically as a diuretic, inhibits Na^+^ channels in renal tubular epithelial cells and is a non-specific inhibitor of ASICs [11]. With an IC50 of ~10-50 μM, amiloride not only suppresses ASIC currents but also prevents upsurges of intracellular Ca^2+^ and plasma membrane depolarization induced by extracellular acidosis. Studies of the Na^+^ ion channel family indicate that amiloride directly blocks ASIC activation, thereby inhibiting current generated via acidic pH levels [24].

A-317567 is a small molecule (reportedly more selective than amiloride) and a nondiscriminatory inhibitor of ASIC currents in sensory and central neurons capable of blocking ASIC1a, ASIC2a, and ASIC3 currents [25]. Unlike amiloride, A-317567 provides significant relief from chronic pain induced by tissue acidification [25].

Latest studies also suggest that non-steroidal anti-inflammatory drugs (NSAIDs) help relieve pain by modulating ASIC activity [26]. NSAIDs directly inhibit inflammation-related sensory neuronal increases in expression and activity of ASIC1a and ASIC3, thus conferring their analgesic effect [27, 28].

Tarantula toxin 1 (psalmotoxin, PcTx1) is another selective inhibitor of ASIC1a [29]. In a heterologous expression system, PcTx1 inhibits ASIC1a-induced current (IC50 ~ 1 nM), with no inhibitory effect on currents induced by voltage-gated ion channels, including some K^+^, Na^+^, and Ca^2+^ channels, and ligand-gated channels [30]. At this concentration, PcTx1 changes the affinity of ASIC1a for H^+^ ions, transforming the channels from resting to inactivated states and increasing their closing times [30, 31].

APETx2 is a selective inhibitor of ASIC3 [32]. Declining extracellular pH triggers a biphasic ASIC3 response: fast depolarization followed by a prolonged refractory period. APETx2 inhibits several subunits (i.e., 1a, 1b, 2b) of ASIC3 but does not affect ASIC1a, ASIC1b, ASIC2a, or ASIC3/2a [33]. APETx2 inhibits ASIC3-generated and sustained window currents evoked at pH 7.0 [34].

The Texas coral snake toxin known as MitTx activates ASIC1a and ASIC1b homomers and enhances pH sensitivity of ASIC2a channels [35]. MitTx has weak effects on ASIC2a if applied at pH 7.4 but potentiates acid-evoked current by shifting the activation curve towards less acidity. The effect of MitTx on ASIC-mediated neuronal currents in trigeminal ganglia of mice is a function of ASIC1a channels and is abolished in ASIC1a-knockout mice [36].

Another agent, 2-guanidine-4-methylquinazoline (GMQ), acts to persistently activate ASIC3 at normal pH [37]. Injection of GMQ into a mouse paw activates ASIC3, triggering pain behavior in wild-type but not in ASIC3-knockout mice. Although the potency of GMQ in activating ASIC3 is low (EC50 ~ 1 mM) at physiologic Ca^2+^ concentrations, GMQ may influence pH dependencies of other ASIC subtypes, preempting current at physiologic pH by a shift to more acidic pH [2].

PhcrTx1 is a compound extracted from the sea anemone (*Phymanthus crucifer*) that inhibits peak neuronal ASIC currents in rat DRG [38].

Endogenous compounds that are active in this setting include spermine, which intensifies ASIC1a activation and aggravates ischemic neuronal injury [39], and agmatine, which activates homomeric ASIC3 and heteromeric ASIC3-ASIC1b [37, 40]. Mambalgin-1, a peptide isolate of snake venom, selectively inhibits currents mediated by ASIC1and ASIC1b homomers and heteromers [41]. The potent protease inhibitor, nafamostat, is used to treat acute pancreatitis [42]. It also inhibits ASIC1a and ASIC2a and blocks initial-phase transient currents of ASIC3 [43].

## ASICs as potential therapeutic targets for treating neurologic diseases

The various functions of ASICs in the nervous system have yet to be fully understood. ASICs are widely distributed throughout all regions of the brain where they participate in synaptic plasticity, learning, and sensory transduction. In particular, ASIC1a is a known purveyor of synaptic plasticity, especially the facilitation of N-methyl-D-aspartate (NMDA) receptor activation in long-term potentiation (LTP) of hippocampus [44]. However, a recent study by the Lien group showed that disruption of the ASIC1a gene did not impair normal hippocampal LTP and spatial memory in ASIC1a conditional knockout mice [45]. Still, Wemmie et al. recently detected local pH changes during normal brain activity in mouse and human brains, underscoring the potential for ASIC activation in this context [46]. More studies are clearly needed to delineate the roles of ASIC1 in the nervous system (Table 2).

**Table 2:** ASICs in neurologic disorders

Disease	Role of ASICs
Parkinson’s disease	Lactic acidosis occurs in the brains of patients with PD.
	Amiloride helps protect against substantia nigra neuronal degeneration, inhibiting apoptosis.
	Parkin gene mutations result in abnormal ASIC currents.
Huntington’s disease	ASIC1 inhibition enhances ubiquitin-proteasome system activity and reduces huntingtin-polyglutamine accumulation.
Pain	ASIC3 is involved in: 1) primary afferent gastrointestinal visceral pain, 2) chemical nociception of the upper gastrointestinal system, and 3) mechanical nociception of the colon.
	Blocking neuronal ASIC1a expression in dorsal root ganglia may confer analgesia.
	NSAIDs inhibit sensory neuronal ASIC expression.
Cerebral ischemia	Neuronal ASIC2 expression in the hypothalamus is upregulated after ischemia.
	Blockade of ASIC1a exerts a neuroprotective effect in a middle cerebral artery occlusion model.
Migraine	Most dural afferent nerves express ASICs.
Multiple sclerosis	ASIC1a is upregulated in oligodendrocytes and in axons of an acute autoimmune encephalomyelitis mouse model, as well as in brain tissue from patients with multiple sclerosis.
	Blockade of ASIC1a may attenuate myelin and neuronal damage in multiple sclerosis.
Seizure	Intraventricular injection of PcTX-1 increases the frequency of tonic-clonic seizures.
	Low-pH stimulation increases ASIC1a inhibitory neuronal currents.
Malignant glioma	ASIC1a is widely expressed in malignant glial cells.
	PcTx1 or ASIC1a knock-down inhibits cell migration and cell-cycle progression in gliomas.
	Amiloride analogue benzamil also produces cell-cycle arrest in glioblastoma.

## Parkinson’s disease

Parkinson’s disease (PD) is a disabling disease characterized by selective, gradual apoptosis of midbrain dopaminergic (DA) neurons and progressive motor deterioration. Increasing efforts have been made to identify putative causes of PD, namely oxidative stress, microglial inflammation, and mitochondrial dysfunction. Interestingly, these processes often result in tissue acidification; and lactic acidosis, which further aggravates neuronal damage, has been documented in the brains of patients with PD and in the classic 1-methyl-4-phenyl-1,2,3,6-tetrahydropyridine (MPTP)-induced animal model of PD [47, 48]. A recently conducted study has demonstrated that mitochondrial ASIC1a may serve as an important regulator of MPT pores, thus contributing to oxidative neuronal cell death [49]. Moreover, Arias and colleagues have found that MPTP-treated mice develop brain tissue acidosis and that ASIC inhibitors amiloride and PcTx-1 protect against substantia nigra neuronal degeneration by reducing levels of DA and its transporter and preventing apoptosis [50]. In addition, mutation of the parkin gene or a lack of endogenous parkin protein results in abnormal ASIC currents, protein degradation, and DA neuronal injury, suggesting that ASIC currents may mediate the fundamental pathology in PD [51]. ASIC inhibitors amiloride and PcTx-1 are also protective of substantia nigra in the mouse PD model, preventing neuronal loss and apoptosis [50]. Lipopolysaccharide (LPS) stimulation likewise increases levels of ASIC1 and ASIC2a expression in rat microglia and induces inflammatory cytokines [52]. Such findings collectively indicate that modulating microglial ASIC function may control neurodegenerative diseases such as PD.

## Huntington’s disease

In individuals with Huntington’s disease (HD), CAG repeats of the huntingtin gene *IT15* (copy number >35–40) produce variations in the huntingtin protein, leading to duplicated glutamine residues. The proteins gradually form large aggregates in the brain, leading to disrupted neuronal function and neuronal death. As yet, there is no effective treatment. However, Wong et al. have reported that ASIC1 inhibition enhances ubiquitin-proteasome system activity and reduces huntingtin-polyglutamine accumulation, implicating ASICs in the pathogenesis of HD as well [53].

## Pain

Tissue acidosis often increases pain sensitivity. Release of H^+^ ions in damaged tissue lowers the pH locally, promoting pain receptor depolarization and generating pain [14]. Somatic pain originating in the peripheral nervous system is attributable to ASIC3 [54]. This not only pertains to primary afferent gastrointestinal visceral pain but also applies to chemical nociception of the upper gastrointestinal system and to mechanical nociception of the colon [55]. Hence, ASIC3 inhibition may be a means of relieving chronic abdominal pain [56]. Analgesia may similarly be achieved by blocking neuronal ASIC1a expression in DRG, so it appears that ASIC1a activation is also a factor in sensitization of the peripheral nervous system and generation of pain [56]. A-317567, an inhibitor of ASIC1/3, alleviates skin pain in mice after surgery [56]. Additionally, NSAIDs may inhibit ASIC expression in sensory neurons, block Ca^2+^ channels, and reduce peak ASIC currents [28, 57]. Expression levels of ASIC1a and ASIC2a are upregulated in the spinal cord, presumably to effect central pain sensitization [58, 59]. Genetic disruption of ASIC1a alleviates mechanical hyperalgesia elicited by intrathecal brain-derived neurotrophic factor (BDNF) injections [60], and intrathecal administration of PcTx-1 mitigates pain behavior in rodents [61]. These studies provide compelling evidence that inhibition of ASICs may ease pain.

## Cerebral ischemia

Severe cerebral ischemia may lower brain pH to 6.3 or less, resulting in Ca^2+^ overload and neuronal cytotoxicity. ASIC1a and ASIC2a are widely expressed in the central nervous system [56]. Because ASIC1a is selectively permeable to Ca^2+^, activation of ASIC1a-bearing channels promotes Ca^2+^ influx [4]. Activation of ASIC1a may trigger membrane depolarization, spurring Ca^2+^ influx directly via ASIC1a homomers or ASIC1a/2b heteromers, voltage-gated Ca^2+^ channels, and NMDA receptors [62]. Intracellular Ca^2+^ overload then evokes a sequence of cytotoxic events that ultimately aggravate tissue and cell damage through intracellular enzyme activation. Cytotoxicity due to acidic metabolites and intracellular Ca^2+^ overload causes protein, lipid, and nucleic acid degradation, with apoptosis and necrosis as eventual endpoints. Zhang et al. found that ischemia has no effect on neuronal ASIC1 expression in the hypothalamus but ASIC2 expression and expression of anti-apoptotic proteins Bcl-2 and Bcl-W were upregulated [63], so perhaps ASIC2 takes part in preventing apoptosis induced by cerebral ischemia [63]. Nevertheless, other studies do show that ASIC1a blockade confers neuroprotective effects in a middle cerebral artery occlusion model [39, 64]. Thus, the functional roles of ASICs may vary in differing pathologic states, rendering them a new therapeutic target in ischemic brain injury.

## Migraine

Migraine is typically a severe unilateral or bilateral pulsatile headache. Studies indicate that ASICs have major roles in activating pain-sensitive afferent nerves during migraines [65, 66], including dural branches [66]. Dural ischemia or release of macrophage granular contents may cause local inflammatory changes, lowering tissue pH locally and in turn activating ASICs and dural primary afferent nerves [67]. Neuronal ASIC3 expression is found in most trigeminal ganglia (TG) and dural afferents [68]. Calcitonin gene-related peptide (CGRP) release, resulting in neurogenic inflammation and headache progression, is increased in TG neurons via ASIC3 activation [68]. In a preclinical study, amiloride was found to block cortical spreading depression (CSD) and inhibit TG activation, implying that an ASIC1-dependent mechanism is operant [69]. ASIC signaling cascades then seem to be critical in development of migraine, despite the need for greater clarity at present.

## Multiple sclerosis

ASIC1a is upregulated in oligodendrocytes and in axons of an acute autoimmune encephalomyelitis mouse model, as well as in brain tissue from patients with multiple sclerosis, and this increased expression is associated with axonal injury [70]. On the other hand, genetic disruption of ASIC1 alleviates this axonal degeneration and reduces clinical deficits [71]. In addition, amiloride attenuates myelin and neuronal damage in multiple sclerosis [72]. Blockade of ASIC1a expression may therefore confer neuroprotective effects in patients with multiple sclerosis.

## Seizure

During seizure activity, intense neuronal firing causes a drop in brain pH, raising the possibility that abnormal activation of ASICs affect onset and maintenance of epileptic seizures. The frequency of tonic-clonic seizures is increased by intraventricular injection of the ASIC1a inhibitor PcTX-1 [73]. However, EEG tracings confirm that ASIC1a overexpression does not affect seizure onset and may prematurely terminate episodes [74]. As opposed to ASIC1a-knockout mice, wild-type mice show declines in magnitude and intensity of seizures, indicating that a drop in pH and augmented ASIC1a expression both signal antiepileptic outcomes [75]. Furthermore, low-pH interneuronal and excitatory pyramidal cell stimulation increases ASIC1a inhibitory neuronal currents, suggesting that ASIC1a may limit seizures by dampering neuronal excitability [75].

## Malignant glioma

ASIC1a is widely expressed in malignant glial cells. Amiloride- and PcTx1-sensitive cation currents have been recorded in human glioblastoma [76], and PcTx1 or ASIC1a knock-down inhibits cell migration and cell-cycle progression in gliomas [77, 78]. ASIC1a inhibition leads to cell-cycle arrest through upregulation of cyclin-dependent kinase inhibitor protein expression and blockade of growth factor phosphorylation cascades [77, 78]. The amiloride analogue, benzamil, also produces cell-cycle arrest in glioblastoma [77]. This body of evidence clearly links ASIC1a activation with cellular growth and migration in malignant gliomas.

## Conclusion

In this review, expression, localization, and mechanisms (biochemical, physiologic, and pathophysiologic) of ASICs are discussed. Based on a substantial body of research, largely derived from animal models, their involvement in various neurologic diseases seems certain. It is therefore important to determine how this knowledge translates to humans, given that ASIC-blocking drugs have may have therapeutic potential in a spectrum of neurologic and psychiatric disorders. Better understanding of related molecular mechanisms and signaling pathways will help clarify the role of ASICs going forward.
